# Public Interest in Preventive Measures of Coronavirus Disease 2019 Associated With Timely Issuance of Statewide Stay-at-Home Orders

**DOI:** 10.1017/dmp.2020.189

**Published:** 2020-06-05

**Authors:** Benjamin Greiner, Ryan Ottwell, Matt Vassar, Micah Hartwell

**Affiliations:** University of Texas Medical Branch, Department of Internal Medicine, Galveston, TX; Oklahoma State University Center for Health Sciences, College of Osteopathic Medicine, Tulsa, OK; Oklahoma State University Center for Health Sciences, College of Osteopathic Medicine, Department of Psychiatry and Behavioral Sciences, Tulsa, OK

**Keywords:** COVID-19, infection control, public awareness, public preparedness

## Abstract

**Introduction:**

One method of monitoring public preparedness is through measuring public interest in preventive measures. The objective of this study was to analyze public interest in the coronavirus disease 2019 (COVID-19) preventive measures and to identify variables associated with timely stay-at-home (SAH) orders issued by governors.

**Methods:**

State-level search volume was collected from Google Trends. Average preventive measure interest was calculated for the query terms “hand sanitizer,” “hand washing,” “social distancing,” and “COVID testing.” We then calculated the delay in statewide SAH orders from March 1, 2020, to the date of issuance and by-state presidential voting percentage. Bivariate correlations were computed to assess the relationship between interest in preventive measures and SAH order delay.

**Results:**

The correlation between average preventive measure interest and length of time before the SAH order was placed was −0.47. Average preventive measure interest was also inversely related to voting for a Republican presidential nominee in the 2016 election (R = −0.75), the latter of which was positively associated with longer delays in SAH orders (R = 0.48).

**Conclusions:**

States with greater public interest in COVID-19 preventive measures were inversely related to governor issuance of timely SAH orders. Increasing public interest in preventive measures may slow the spread of the virus that causes COVID-19, severe acute respiratory syndrome coronavirus 2 (SARS-CoV-2), by improving preparedness.

The emergence of novel infectious diseases, such as severe acute respiratory syndrome (SARS) and H1N1 influenza pandemic, has consistently exposed the socioeconomic, physical, and preparedness weaknesses in modern society.^1^ For instance, 1 study analyzed the financial impact of a mild influenza pandemic on the global economy, which estimated a cost of close to 0.8% of gross domestic product, which was equivalent to US $330 billion.^2^ Fortunately, global trends of emerging infectious diseases have been strongly correlated with several identifiable factors – human density and the distribution of wildlife biodiversity.^1^ Thus, the targeting of surveillance efforts to specific “hotspots,” using spatially explicit models, can allow for better allocation of resources and preparation for disease outbreaks.^1^ However, preparation for outbreaks continues to present challenges that contribute to the negative socioeconomic and health effects of pandemics.

In the wake of recurrent pandemics in the 19th and 20th centuries, the World Health Organization (WHO) published its first pandemic plan in 1999, which focused primarily on surveillance, yet minimally discussed plans for response or communication efforts that are necessary for pandemics.^3^ In response to the 2002–2004 SARS pandemic, the Department of Health and Human Services and White House strategy for a pandemic response ultimately recognized the need for increased planning efforts, which resulted in the 2006 Pandemic and All-Hazards Preparedness Act – calling for the critical review of state pandemic preparedness plans.^4^ All combined efforts fundamentally led to the uptake of the 4 pillars of pandemic response: surveillance, vaccine and antiviral development, emergency response, and communication.^5^

Although the need for local and state-based pandemic response plans has long been recognized as essential,^4^ many communities continue to lack pandemic preparedness. One study indicated that only 17 states (35%) explicitly discussed the use of voluntary self-isolation and only 15 states (31%) discussed the use of institutional or household quarantine as measures for reducing the morbidity and mortality of pandemics.^6^ Likewise, the link between partisan interests and the coronavirus disease 2019 (COVID-19) outbreak has recently come to fruition as Republican voters reported less concern.^7^ The current pandemic outbreak of COVID-19, a result of the virus, severe acute respiratory syndrome coronavirus 2 (SARS-CoV-2), offers a new opportunity to evaluate the pandemic response preparedness of US states. Following the 2014–2015 Ebola epidemic in Sierra Leone, West Africa, a recent study found that public awareness and knowledge of Ebola were high, yet awareness of preventive measures was lacking, which likely impacted the transmission rate.^8^ Therefore, we posit that 1 method in monitoring preparedness during the COVID-19 pandemic is through the analysis of public interest in prevention techniques. Thus, the objective of this study was to analyze public interest in COVID-19 preventive measures and to identify and describe variables that were associated with timely stay-at-home (SAH) orders issued by state governors.

## METHODS

### Study Sample and Measures

We collected state-level search volume data using Google Trends (https://trends.google.com/trends/). In this study, we defined public interest as the scaled search volume as computed by Google Trends. Google Trends was used to analyze relative interest because it is the most widely used search engine for obtaining health information by patients.^9,10^ The regional volumes from Google Trends are scaled from 0 to 100, with 100 being the greatest proportion of queries (POQ) among all searches conducted in that region over the specified time period. We used search topics related to prescribed preventive measures recommended by the surgeon general and the US President’s medical council and conducted the searches on April 6, 2020, over the time period of March 1 through April 5, 2020. These terms included “hand sanitizer,” “hand washing,” “social distancing,” and “COVID testing,” and we also searched for “COVID vaccine,” “azithromycin,” and “hydroxychloroquine,” because they were prominently featured in the President’s daily COVID-19 press briefings. The average of preventive measures was calculated for “hand sanitizer,” “hand washing,” “social distancing,” and “COVID testing” POQ per state.

Additionally, we compiled the dates of statewide SAH orders issued by governors and limitations placed on social and group gatherings, number of confirmed cases and deaths^11^ through April 6, 2020, and by-state percentage of votes cast for a Republican nominee in the 2016 presidential election. We calculated the delay in SAH orders as the length of time in days from March 1, 2020, to the date that the order was issued. States without SAH orders were assigned a length of days of 45, 10 days longer than the nearest length of delay. We analyzed delay in SAH orders by individuals and Republican voting opposed to Democratic voting predominance, as recent literature suggests that the former search less for information on COVID-19, engage in less social distancing behavior, and have a lower perception of risk.^7^

### Statistical analysis

We computed bivariate correlations using the compiled and computed variables to assess the relationship between public interest in measures to prevent the spread of COVID-19, the delay of SAH orders, and the political leaning of the state. Analyses were conducted using Stata 16.1 (StataCorp, College Station, TX).

## RESULTS

**FIGURE 1:**
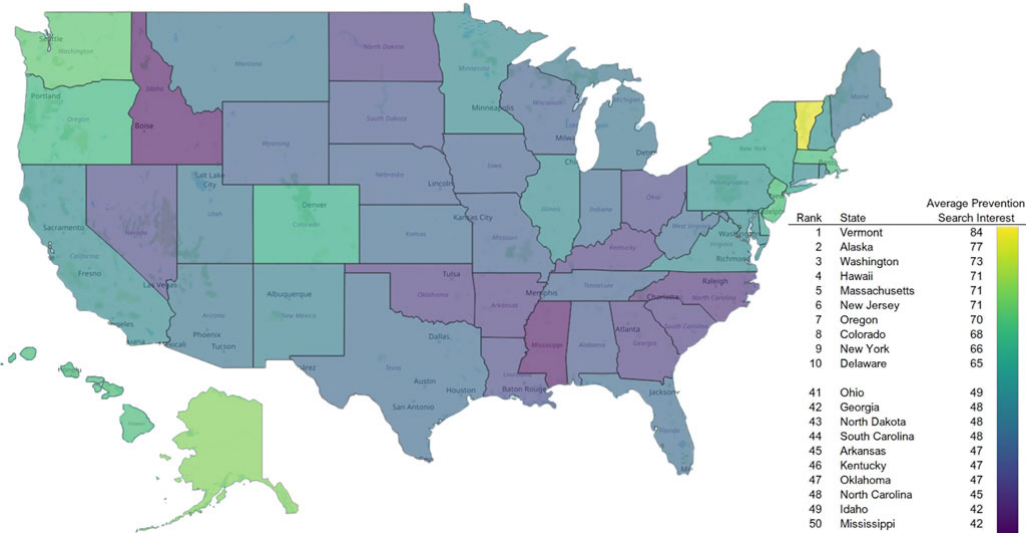
Average COVID-19 Prevention Search Interest

Our results show that states with the highest POQ in COVID-19 prevention were also states that had the quickest response from the governors to order their constituents to stay at home; the correlation between average preventive measures and length of time before an SAH order was placed was −0.47 (Table 1). These states were also inversely related to voting for a Republican presidential nominee in the 2016 election (R = −0.75). These findings indicate that by-state Republican voting was strongly correlated with longer delays in governor issuance of SAH orders. Further, the statewide voting percentage for a Republican presidential nominee in the 2016 election was inversely associated with POQ in any preventive measures, however, was positively associated with searches for hydroxychloroquine (R = 0.27) or azithromycin (R = 0.49) and was associated with longer delays in SAH orders (R = 0.48). Statewide average search interest in preventive measures is represented in Figure 1.

**TABLE 1:** Correlations of Preventive Measures Associated With COVID-19, Delay in Issuance of Stay-at-Home Orders, and Other Variables of Interest

	Average of Preventive Measures^a^	Hand Sanitizing	Hand-washing	Social Distancing	COVID-19 Testing	Stay-at-Home Order^b^	Limited Gatherings^c^	Republican Voting (%)^d^	Azithromycin
Hand sanitizing	0.56								
Handwashing	0.73	0.08							
Social distancing	0.78	0.16	0.70						
COVID-19 testing	0.71	0.50	0.17	0.27					
Stay-at-home order^b^	−0.47	−0.51	−0.26	−0.23	−0.37				
Limited gatherings^c^	−0.13	−0.21	0.06	−0.04	−0.19	0.53			
Republican voting (%)^d^	−0.75	−0.64	−0.40	−0.62	−0.49	0.48	0.19		
Azithromycin	−0.52	−0.08	−0.52	−0.76	−0.08	0.25	0.01	0.49	
Hydroxychloroquine	−0.20	0.02	−0.34	−0.32	0.10	0.02	−0.15	0.27	0.4339

a Average interest in preventive measures (hand sanitizing, Handwashing, COVID-19 testing, and social distancing).

b Days since March 1, 2020, stay-at-home order was issued.

c Limited gatherings were rated based on level of restriction: bans on *all* gatherings = 0; 10 or more persons = 1; 25 or more = 25; 50 or more = 3.

d Republican voting (%) from the 2016 presidential election results.

## DISCUSSION

Public health preparedness for emerging infectious disease outbreaks is 1 of the most effective and necessary methods of infection control, yet the current COVID-19 pandemic brings current preventive practices into question. We identified several variables associated with quicker SAH orders. Lower state-based interest in preventive measures, such as social distancing, hand washing, hand sanitizing, COVID-19 vaccinations, and COVID-19 testing was associated with longer delays in state governors’ SAH orders. Considering that quarantines are essential in highly infectious disease outbreaks, our findings support the need for swift federal and state action, including the closure of non-essential operations, even insituations where constituencies may lack interest in preventive measures for the health of all.

Our research supports that politicians’, in this instance, state governors’, decisions are aligned with the preferences of their voters. Interestingly, studies show that elected officials change their behaviors toward the constituency of their voters independently of the politicians’ personal characteristics and beliefs.^12^ This could have dangerous outcomes, including prolonged spread of the virus, increased morbidity and mortality, and worsening of personal protective equipment shortages, as governors of Republican-voting states may delay SAH orders, against recommendations from the Centers for Disease Control (CDC) and WHO, to appeal to the interest of their voters. For example, the Tennessee (voted a Republican presidential nominee in the 2016 election) state governor delayed the SAH order for 6 days compared with its politically opposite neighbor, Kentucky. As a result, Tennessee had a significantly high number of COVID-19 confirmed cases as compared with Kentucky.^13^ Therefore, we recommend that governors follow recommendations from evidence-based organizations, such as the CDC and WHO, when making critical decisions regarding SAH orders rather than considering political fallout or their state’s interest in such topics.

## CONCLUSIONS

We identified several variables associated with the timely issuance of SAH orders. Notably, states with greater public interest in COVID-19 preventive measures were inversely related to governor issuance of timely SAH orders. Thus, increasing public interest in preventive measures may slow the spread of SARS-CoV-2 by improving knowledge and preparedness as demonstrated in other outbreaks,^14^ in this case, through appropriate handwashing, social distancing, and hand sanitizing. Stakeholders should make every effort to increase public interest in COVID-19 preventive measures.

## References

[1] Keesing F , Belden LK , Daszak P , et al. Impacts of biodiversity on the emergence and transmission of infectious diseases. Nature. 2010;468(7324):647-652. 21124449 10.1038/nature09575 PMC7094913

[2] Centre for Applied Macroeconomic Analysis, Crawford School of Public Policy, Australian National University. Global macroeconomic consequences of pandemic influenza. February 2006. https://cama.crawford.anu.edu.au/pdf/working-papers/2006/262006.pdf. Accessed April 8, 2020.

[3] Gust ID , Hampson AW , Lavanchy D . Planning for the next pandemic of influenza. Rev Med Virol. 2001;11(1):59-70. 11241802 10.1002/rmv.301

[4] Iskander J , Strikas RA , Gensheimer KF , et al. Pandemic influenza planning, United States, 1978–2008. Emerg Infect Dis. 2013;19(6):879-885. doi:10.3201/eid1906.121478. 23731839 PMC3713824

[5] Gensheimer KF , Fukuda K , Brammer L , et al. Preparing for pandemic influenza: the need for enhanced surveillance. Vaccine. 2002;20(Suppl 2):S63-S65. 12110262 10.1016/s0264-410x(02)00135-4

[6] Holmberg SD , Layton CM , Ghneim GS , Wagener DK . State plans for containment of pandemic influenza. Emerg Infect Dis. 2006;12(9):1414-1417. 17073091 10.3201/eid1209.060369 PMC3294751

[7] Barrios J , Hochberg Y . Risk perception through the lens of politics in the time of the COVID-19 pandemic. NBER Working Paper Series, #27008. 2020. doi:10.3386/w27008.

[8] Jalloh MF , Sengeh P , Monasch R , et al. National survey of Ebola-related knowledge, attitudes and practices before the outbreak peak in Sierra Leone: August 2014. BMJ Glob Health. 2017;2(4):e000285. 10.1136/bmjgh-2017-000285 PMC5728302 29259820

[9] Chang K , Berthelet E , Grubbs E , et al. Websites, websites everywhere: how thyroid cancer patients use the Internet. J Cancer Educ. 2019. doi:10.1007/s13187-019-01576-5. 31332622

[10] Jamnadass E , Aboumarzouk O , Kallidonis P , et al. The role of social media and Internet search engines in information provision and dissemination to patients with kidney stone disease: a systematic review from European Association of Urologists Young Academic Urologists. J Endourol. 2018;32(8):673-684. 29926740 10.1089/end.2018.0319

[11] Johns Hopkins University & Medicine. Coronavirus Resource Center. 2020. https://coronavirus.jhu.edu/. Accessed April 7, 2020.

[12] Alesina A . Credibility and policy convergence in a two-party system with rational voters. Am Econ Rev. 1988;78(4):796-805.

[13] A natural Coronavirus experiment is playing out in Kentucky and Tennessee. BuzzFeed News. https://www.buzzfeednews.com/article/danvergano/coronavirus-kentucky-tennessee-social-distancing. Accessed April 13, 2020.

[14] Hasanov E , Zeynalova S , Geleishvili M , et al. Assessing the impact of public education on a preventable zoonotic disease: rabies. Epidemiol Infect. 2018;146(2):227-235. 29271331 10.1017/S0950268817002850 PMC5851038

